# Rapid Detection of Dimethoate in Soybean Samples by Microfluidic Paper Chips Based on Oil-Soluble CdSe Quantum Dots

**DOI:** 10.3390/foods10112810

**Published:** 2021-11-15

**Authors:** Xinpeng Yan, Zhong Zhang, Runguang Zhang, Tian Yang, Guoying Hao, Li Yuan, Xingbin Yang

**Affiliations:** 1 Shaanxi Engineering Laboratory for Food Green Processing and Safety Control, Engineering Research Center of High Value Utilization of Western Fruit Resources, Ministry of Education, College of Food Engineering and Nutritional Science, Shaanxi Normal University, Xi’an 710062, China; echoyan@snnu.edu.cn (X.Y.); zzhang@snnu.edu.cn (Z.Z.); tianyang@snnu.edu.cn (T.Y.); haogy@snnu.edu.cn (G.H.); Yuanli112086@snnu.edu.cn (L.Y.); xbyang@snnu.edu.cn (X.Y.); 2 Xi’an Key Laboratory of Characteristic Fruit Storage and Preservation, Shaanxi Key Laboratory for Hazard Factors Assessment in Processing and Storage of Agricultural Products, Shaanxi Normal University, Xi’an 710119, China

**Keywords:** oil-soluble CdSe QDs, dimethoate, microfluidic paper chip, fluorescence sensor

## Abstract

Given the imperative of monitoring organophosphorus pesticides (OPs) residues in the ecosystem, here a novel, facile and sensitive fluorescence sensor is presented for the rapid detection of dimethoate. In this work, surface molecularly imprinted polymer (SMIP) and microfluidic technology had been introduced to enhance the selectivity and portability of the described methodology. Oil-soluble CdSe quantum dots (QDs) synthesized in a green way were used as fluorescent material for the selective detection of dimethoate on the basis of static quenching and photoinduced electron transfer mechanism. Among many kinds of paper materials, glass fiber paper was used as the novel substrate of paper chip due to low pristine fluorescence and better performance when combining CdSe QDs. In the process of molecular imprinting, the interaction between several functional monomers and dimethoate molecule was investigated and simulated theoretically by software to improve the selectivity of the sensor. Consequently, the fabricated novel detection platform could effectively respond to dimethoate in 10 min with the concentration range of 0.45–80 μmol/L and detection limit of 0.13 μmol/L. The recovery in the spiked experiment soybean sample was in an acceptable range (97.6–104.1%) and the accuracy was verified by gas chromatography-mass spectrometry, which signified the feasibility and potential in food sampling.

## 1. Introduction

For decades, organophosphorus pesticides (OPs) have been widely used because of their excellent ability in the control of plant diseases and insect, which have contributed greatly to the prosperity and progress of agriculture [1]. However, the uncontrolled application of organophosphorus pesticides will cause their massive existence and long-term accumulation in the ecosystem, such as in agricultural products, water, soil, and so forth, causing serious damage to the natural environment. At the same time, the health of human and animals is also at risk. In this regard, organophosphorus pesticides can inhibit the activity of acetylcholinesterase, leading to the accumulation of acetylcholine and, finally, interferes with the normal transmission of the nervous system. To make matters worse, this inhibition is irreversible [2]. Thus, acute and repeated exposures to OPs may cause a host of serious health problems including memory loss, anxiety, depression, psychotic symptoms, deficits in attention and information processing and even possibly coma or death [3]. One such common organophosphorus pesticide is dimethoate which is widely used in the cultivation of fruit and grains. Besides its cholinesterase inhibiting effect, dimethoate can be converted to omethoate, its oxon-derivative, which has higher neurotoxicity [4]. Therefore, it is particularly important to control and monitor the OPs in the environment. Currently, methods used to detect OPs include gas chromatography (GC) [5], high performance liquid chromatography (HPLC), electrochemical methods [6] and biosensors [7], etc. While most of these methods with high precision and sensitivity can meet the requests for trace analysis of pesticide residue, there are still some shortcomings that cannot be ignored, such as complex operation, high cost of time and instrumentation, and need for professional labor. For example, as the most widely used method for detecting OPs, GC usually requires a complicated and lengthy pretreatment process including extraction, purification, concentration, etc., and the results are subject to the matrix effect. As for various electrochemical methods and biosensors, their reproducibility is poor. These facts provide an opportunity for the emergence of more convenient, economical and accurate detection methods.

To date, fluorescence sensors exhibit many merits such as high sensitivity, good accuracy, and lower detection limits [8]. At the same time, the introduction of quantum dots (QDs) with excellent fluorescence performance (e.g., high luminous intensity, good stability, narrow emission spectrum, tunable mission, etc.) have taken the fluorescence sensors to a new height, among which semiconductor QDs such as CdSe, ZnSe, CdTe, CdSe-ZnS, and Mn-doped ZnS play an important role and are widely used [9]. The detection mechanism of fluorescence sensors using QDs is generally based on fluorescence quenching or enhancement after introducing the target. Nevertheless, it cannot be denied that in practice this kind of sensor will be more or less interfered with by coexisting substances that generate fluorescent signals similar to the target, resulting in low selectivity and sensitivity [10]. Thus, the combination with other technologies characterized by high selectivity will further expand the application scope of fluorescence sensors.

As a kind of novel technology with good selectivity, anti-interference ability, as well as low cost and ease of synthesis, molecularly imprinted polymers (MIPs) have been attracting more and more attention [11]. However, MIPs also have some inevitable shortcomings, such as the varying degrees of template molecular residues, weak binding force, slow mass transfer rate and so on, which hampers the widespread adoption of MIPs [12]. Accordingly, references about the applications of surface molecularly imprinted polymers (SMIPs) have been emerging, especially for the detection of some common harmful substances, including but not limited to pesticides [13,14], fungal toxin. Generally, there are three types of interaction between templates and functional monomers, including covalent, non-covalent, and semi-covalent interactions, among which the non-covalent bond (including hydrogen bonding, electrostatic interaction, hydrophobic interaction, metal chelation, coordination, etc.) is the most widely adopted [15]. In the process of synthesis, the performance of MIPs is affected by many factors, such as the type and number of crosslinking agents, functional monomers and reaction solvent [16]. Hence, the interaction between template molecules and functional monomers with different types and amounts should be studied in order to obtain MIPs with better performance.

Microfluidic paper-based analytical devices (μPADs, also referred to as microfluidic paper chips) are the hotspot of current research. Compared with other sensors, μPADs have many advantages, such as inexpensive cost, small size, convenient storage and transportation, consumption of a low volume of reagents, ease of mass production and above all, they are able to take effect without external force [17,18]. At present, they are widely used in many fields from medical analysis and environmental monitoring to disease diagnosis [19].

Based on the above discussion, we focused on the advantages of oil-soluble QDs (e.g., good crystal structure, narrow size distribution and stable fluorescence performance) and constructed a novel rapid detection platform. Taking dimethoate as the target, the sensor could broaden the application range of paper chips. Besides, the incorporation of SMIPs ensured that the detection platform had better selectivity and accuracy. Additionally, the interaction between different functional monomers and template molecules was studied to enhance the recognition ability of MIPs. Materials Studio 2019 software was innovatively used to provide theoretical support for the molecular imprinting process. To sum up, the novel detection platform had many attractive features: portable, user-friendly, economical, and disposable. More importantly, the response time of paper chips to template molecules was only 10 min, which met the requirement of rapid detection.

## 2. Materials and Methods

### 2.1. Materials

The used substrate papers, glass fiber film (SB08, CB06, BT53, RB45), polyester fiber film (VL78), and special absorbent paper (SX18) were purchased from Shanghai Jinbiao Biological Technology Co., Ltd. (Shanghai, China), cadmium oxide, selenium powder, liquid paraffin, oleic acid, oleyl-amine, tetra-methoxy-silane(TMOS), tetra-ethoxy-silane (TEOS), tetra-propoxy-silane (TPOS), 3-aminopropyltriethoxylsilane (APTES), trimethoxy-silyl-propane-thiol (MPTMS), and vinyl-trimethoxy-silane(VMS) were supplied by Aladdin (Shanghai, China); urea, sodium dodecyl sulfonate (SDS), sodium chloride, trichlorfon, acephate, fenthion and dimethoate were supplied by Sigma-Aldrich (Shanghai, China). Soybean samples were purchased from a local market in Xi’an (Shaanxi, China). All the water used in the experiment was ultrapure. All reagents used were of at least analytical grade.

### 2.2. Synthesis of CdSe QDs

According to the previously published method [20], CdSe QDs were synthesized in a cost-effective and environmentally friendly manner by appropriately modifying the reaction time and temperature. The reaction was carried out under the protection of nitrogen, and when the reaction stopped, the mixture was cooled to room temperature, then washed with methanol to precipitate CdSe QDs, and centrifuged at 9000× *g* for 10 min. After several repeated extraction, CdSe QDs were dispersed in dichloromethane and stored in the dark at 4 °C for later use. The whole formation process of CdSe QDs can be summarized in the following equations, where Equation (1) was the general reaction equation [20].

(1) $$\mathrm{CdO}\;+\;\mathrm{acid}\;+\;\mathrm{Se}\left(\mathrm{oxidant}\right)\overset{△}{\to }\;\mathrm{CdSe}\;+\;\mathrm{oxided}\;\mathrm{products}$$

(2) $$\mathrm{CdO}+\mathrm{Oleic}\;\mathrm{acid}\;\overset{△}{\to }\;\mathrm{Cd}-\mathrm{complex}$$

(3) $$\frac{n}{4}\mathrm{Se}\;+\;R-{\mathrm{CH}}_{2}\left({\mathrm{CH}}_{2}\right){\mathrm{nCH}}_{3}\overset{\mathrm{Dehydrogenation}}{\to }{\;R-{\mathrm{CH}}_{2}({\mathrm{CH}}_{2}\mathrm{CH}={\mathrm{CHCH}}_{2})\;}_{\frac{n}{4}}{\mathrm{CH}}_{3}+\frac{n}{4}H_{2}\mathrm{Se}$$

(4) $$H_{2}\mathrm{Se}\;+\;\mathrm{Cd}-\mathrm{complex}\overset{△}{\to }\;\mathrm{CdSe}$$

### 2.3. Characterization of CdSe QDs

The fluorescence intensity of CdSe QDs was measured by fluorescence spectrophotometer (F-7000, Hitachi, Tokyo, Japan). The ultraviolet and visible (UV-vis) absorption spectra were recorded with a UV-Visible Spectroscopy (U-3900, Hitachi, Tokyo, Japan). The morphology of CdSe QDs was characterized by transmission electron microscopy (TEM), and high-resolution TEM (HRTEM) was performed on field emission transmission electron microscope (JEM-2800, JEOL, Tokyo, Japan) operating at 200 kV. X-ray powder diffraction (D8 Advance, Brook, Karlsruhe, Germany) was employed to obtain the XRD pattern of CdSe QDs. The test conditions were as follows: Cu target Kα radiation (λ = 0.154 nm), scanning step size 0.02, scanning rate 0.02 sec/step, test interval 10°–60° (2θ).

### 2.4. Construction of a Rapid Detection Device for Paper Chip

#### 2.4.1. Preparation of paper@QDs

After washing with deionized water and drying, the square fiberglass paper (4–6 sheets of 1.0 cm × 1.0 cm) were put into petri dish. 15 mL CdSe QDs solution was added, then oscillated for 12 h at room temperature in darkness. Next, 60 μL TEOS was added as protective material. In order to keep the fluorescent stability in the process of synthesis, it is necessary to control the reaction surface of the paper chip always facing upward.

#### 2.4.2. Preparation of paper@QDs@MIPs

Twelve mg dimethoate and 194 μL MPTMS (the molar ratio was about 1:20) were added to 10 mL ethanol solution and left overnight at 4 °C in darkness (for more than 20 h). After the reaction, 100 μL NH_3_·H_2_O and 50 μL TEOS were added and mixed well. Then the prepared paper@QDs was immersed in the solution subsequently and reacted for 4 h. Finally, the prepared paper chips were washed three times with methanol/HAc mixture (8:2, *v*/*v*) to remove the template dimethoate, then washed with methanol alone to obtain the paper@QDs@MIPs (hereinafter referred to as MIPs). The non-imprinted polymer paper@QDs@NIPs (hereinafter referred to as NIPs) were synthesized under the same conditions without the addition of template dimethoate.

#### 2.4.3. Selection of Paper Substrates

To investigate the combination ability of different paper substrates with CdSe QDs, firstly the background fluorescence intensity of six different paper substrates, including glass fiber film (SB08, CB06, BT53, RB45), polyester fiber film (VL78) and special absorbent paper (SX18), was determined. Next, 15 mL of CdSe QDs, oscillating, were incubated with aforesaid different papers in petri dish overnight in darkness. The paper substrates were then eluted with 8 mL of methanol and 2 mL of 0.01 mol/L acetic acid for 1 h and the fluorescence intensity of paper substrates before and after elution was measured.

#### 2.4.4. Investigation of Binding Forces

According to the previously reference [21], paper@QDs (CB06) were treated with 10 mL 0.1 mol/L urea, 0.1% SDS, 0.2 mol/L NaCl solution and ultrapure water for 30 min, respectively. Each group contained three parallel sheets of paper. After the reaction, the fluorescence intensity of paper substrates before and after the treatment was recorded.

#### 2.4.5. Selection of Functional Monomers

Using ethanol as solvent, the concentration of dimethoate was fixed at 60 μM. The mixed solution of dimethoate and TMOS, TPOS, MPTMS, VMS and APTES with a molar ratio of 1:100 was prepared and placed in 10 mL centrifugal tubes. The liquid was fully mixed by shaking for 30 min, and then stood at 4 °C for 12 h to make the dimethoate and silanization reagents interact fully. With the corresponding concentrations of silanization reagent-ethanol solutions as the reference solutions, the effects of different silanization reagents on UV-vis absorption spectra of dimethoate were measured and after the addition of silanization reagents, the ratio of the absorption peak to the initial peak was recorded.

Afterwards, the mixed solutions with different molar ratio of dimethoate and APTES were prepared, and the corresponding concentrations of APTES–ethanol solutions were used as the reference solutions. The absorption spectra of dimethoate solutions with different concentrations of APTES were recorded. Simultaneously, APTES was replaced with MPTMS, and the above steps were repeated to observe the effects of different concentrations of MPTMS on the absorption spectra of dimethoate.

### 2.5. Characterization of Imprinted Paper Chip

The surface morphology of paper chip in different periods were performed under 20 kV by environmental scanning electron microscope (SEM, Quanta 200, FEI company, Hillsboro, OR, USA). The element scanning and mapping analysis (Zeiss Smart EDX, Carl Zeiss AG, Oberkochen, Germany) was used to verify the existence of the added elements to prove successful modification. Infrared spectra were obtained using an infrared spectrometer (FT-IR, Tensor27, Brook, Karlsruhe, Germany) to determine changes in chemical groups during synthesis. The thermal stability of synthesized MIPs was characterized by a thermal analysis system (10 °C/min, N_2_) (TG, Q1000DSC + LNCS + FACS Q600SDT, TA Instrument, New Castle, DE, USA). In order to further evaluate the fluorescence stability of MIPs, we recorded the fluorescence intensity after multiple excitations within 2 h. All of the fluorescence spectra were detected by fluorescence spectrophotometer (F-7000, Hitachi).

Determination of response time: 30 μL of 60 μM template solution was added to MIPs. Then the fluorescence intensity (λ_ex_ = 370 nm) was measured every 5 or 10 min, and the response time was observed for 1 h.

Titration experiment: dimethoate was added to MIPs and NIPs according to the concentration gradient of 0, 5, 10, 20, 40, 60, 80, 100, 120, 150 μM, respectively, and the fluorescence intensity were determined after reaction for 10 min. In addition, a laser confocal microscope (FV1200, OLYMPUS, Tokyo, Japan) was used to observe the changes in fluorescence intensity of MIPs when adding different concentrations of dimethoate.

### 2.6. Sensitivity and Validation of Method

Determination of selective ability: trichlofon, acephate and fenthion, structural analogues of dimethoate, were selected to form a 100 μM solution. Next, 30 μL were added to MIPs and NIPs, respectively, and the fluorescence intensity of MIPs and NIPs was measured after 10 min reaction. The selectivity of MIPs was investigated by comparing the fluorescence intensity changes before and after adding pesticide.

**Figure 1:**
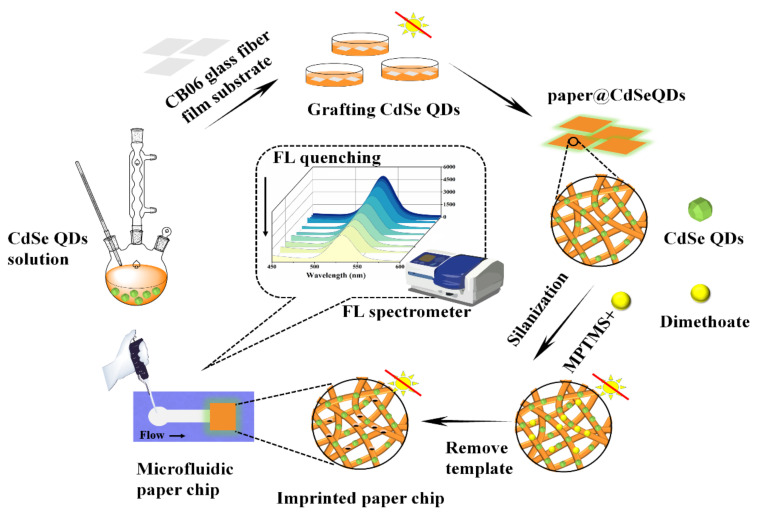
Fabrication diagram of the microfluidic paper chip.

Detection of dimethoate in practical samples: soybean samples were soaked in deionized water for 30 min before treatment. 10 g soaked soybeans were squeezed into juice and filtered with gauze, then the filtrate was placed in centrifuge tubes and centrifuged at 5000× *g* for 15 min. 20 mL ethyl acetate and 5 g anhydrous sodium sulfate were added to the supernatant. After eddy mixing, extraction was carried out. The extraction solutions were then blown to nearly dry with nitrogen at 40 °C and the certain concentrations of standard solutions were added. The concentration of dimethoate in the samples were 0, 5, 10 and 20 μmol/L, respectively. Finally, the solutions were filtered with 0.45 μm filter membrane. 30 μL of aforesaid solutions was transferred into the detection area of the microfluidic paper chip and then the fluorescence intensity was measured. The synthesis process of microfluidic paper chip was shown in Figure 1.

Meanwhile, the labeled samples were detected by gas chromatography-tandem mass spectrometry (GC-MS) to evaluate the accuracy of the proposed method. The detection conditions were as follows: Agilent HP-5 MS column (30 m 0.25 mm, 0.25 μm, inner diameter), and used helium as the carrier gas at 1.69 mL/min. Injection was made without a split ratio at an injection volume of 1 μL and a temperature of 250 °C. The column temperature was initially 50 °C and held for 1 min, then increased to 125 °C at 25 °C/min, finally to 300 °C at 10 °C/min, and this temperature was maintained for 5 min. The electron energy was 70 eV, and the temperature of the ion source was set at 200 °C.

### 2.7. Investigation on Possible Quenching Mechanism

In order to study the possible quenching mechanism of CdSe QDs, the transient fluorescence spectrometer (FLS1000, Edinburgh Instruments, Edinburgh, England) was used to determine the fluorescence lifetimes of CdSe QDs before and after adding 60 μM dimethoate solution. The UV-vis absorption spectra of CdSe QDs were recorded at the same time.

Photolysis experiment: CdSe QDs solution treated with and without Cu^2+^ were prepared and irradiated by ZWF three-use ultraviolet analyzer for 0, 30, 60 and 90 min, respectively, and the UV-vis absorption spectra were recorded.

### 2.8. Statistical Analysis

The analysis of variance (ANOVA) of the data was used to evaluate the significance in the difference between two methods by the SPSS21 (SPSS Inc., Chicago, IL, USA), and *p* < 0.05 was considered statistically significant.

## 3. Results

### 3.1. Characterization of CdSe QDs

**Figure 2:**
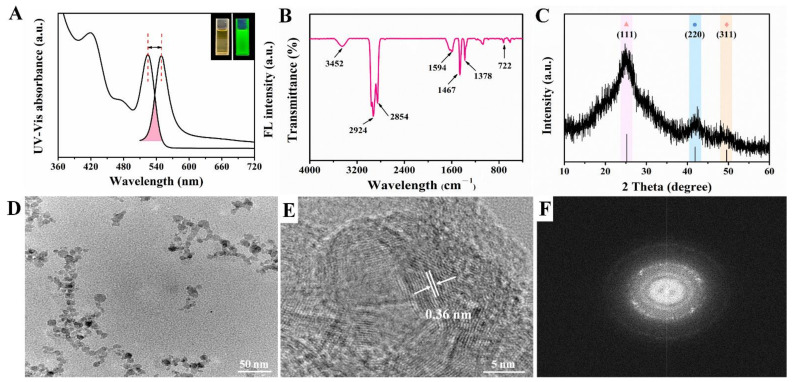
Characterization of oil-soluble CdSe QDs (**A**) Fluorescence spectrum and UV-vis absorption spectrum; the upper right inset showed the photos taken under sunlight and ultraviolet light separately. (**B**) FT-IR spectrum. (**C**) XRD pattern. (**D**) TEM image. (**E**) HR-TEM image. (**F**) SADE pattern.

In this work, CdSe QDs were synthesized by using inexpensive and green reagents. CdO powder and Se powder were used as Cd source and Se source, separately. Paraffin liquid was chosen as the solvent because it was cheaper, more stable and more environmentally friendly than the reported TOPO or ODE solvents [20]. As can be seen from Figure 2A, the upper right inset shows that the synthesized CdSe QDs solution was a homogeneous liquid with pale orange color under the sunlight while exhibiting a bright green fluorescence under the ultraviolet lamp. Stokes shift is the difference between the fluorescence emission peak and the first exciton absorption peak in the ultraviolet spectrum [22]. By Gaussian fitting, the first exciton absorption peak of CdSe QDs was 524 nm and the emission peak of the fluorescence spectrum was 550 nm, respectively. Consequently, the Stokes shift of synthesized CdSe QDs was 26 nm, indicating that it had well-defined structure and minor surface defects [23]. Besides, the full width at half maximum (FWHM) was about 30.5 nm, which represented that the size distribution of CdSe QDs was relatively uniform.

The functional groups of synthesized CdSe QDs were studied by FT-IR, as shown in Figure 2B; 3452 cm^−1^ was the N-H stretching vibration peak, and 1594 cm^−1^ was the coupling peak of N-H bending vibration and C-N stretching vibration, indicating that the main group on CdSe QDs surface was amino. In addition, the absorption peak at 2854 cm^−1^ and 2924 cm^−1^ was the C-H stretching vibration peak, 1378 cm^−1^ was the C-H bending vibration peak of alkane, and 722 cm^−1^ was the characteristic vibration peak of Cd-Se [24].

Due to the small size of QDs, the diffraction peaks were broadened to some extent compared with the bulk materials. The powder XRD pattern of CdSe QDs is shown in Figure 2C. These diffraction features peaks appeared at about 24.79°, 42.16°, and 49.70°, which were in line with the (111), (220), and (311) crystal planes of the zinc-blende phase of CdSe (Joint Committee on Powder Diffraction Standards file No. 19-0191). No other impurity peaks were seen. The FWHM was 6.80° after Gaussian fitting for (111) crystal planes. According to Bragg’s Law (5), the lattice spacing was calculated to be about 0.36 nm. TEM images showed slight aggregation of CdSe QDs, which may be caused by repeated centrifugation and washing by ethanol during sample preparation. According to Figure 2D, the particle size of CdSe QDs was less than 10 nm, which was consistent with the conventional size of QDs reported in the literature [25]. Furthermore, the lattice spacing in HRTEM image (Figure 2E) was 0.36 nm and the selected area electron diffraction (SAED) pattern of CdSe QDs (Figure 2F) was of a typical polycrystalline structure, which agreed well with the XRD results [26]. To sum up, the above results confirmed the good crystallinity and fluorescence properties of CdSe QDs.

D_(111)_ = λ/2sinθ_(111)_ (5)

### 3.2. Selection of Paper Substrates

**Figure 3:**
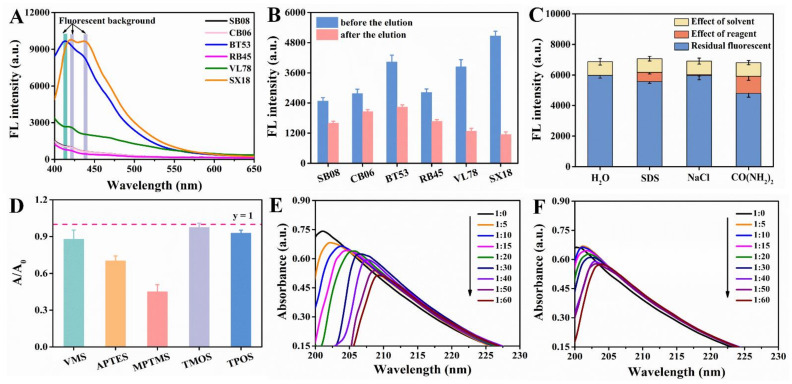
(**A**) Background fluorescence spectra of six different unprocessed papers (SB08, CB06, BT53, RB45, VL78, SX18). (**B**) Fluorescence intensity changes of six different papers grafted with CdSe QDs before and after elution. (**C**) Possible binding mechanism between CB06 and CdSe QDs. (**D**) Absorbance intensity changes of dimethoate before and after reaction with different kinds of silanization reagents (the molar ratio of dimethoate to silanization reagent was 1:100). The absorption spectra of dimethoate treated with different concentrations (molar ratio) of MPTMS (**E**) and APTES (**F**).

Paper substrate was the core component of the entire microfluidic paper chip. The selection of paper substrates was very critical, and multiple factors needed to be considered [27], such as sufficient mechanical endurance; no significant deformation and disintegration when soaked in water phase; the ability to form a clear detection area, but also to avoid excessive dispersion [28]; reduction of the coffee-ring effect that was easy in conventional methods [29], etc. Usually, filter and chromatography papers and nitrocellulose membranes are widely used for µPADs [30]. There are also other types of substrates, such as glass fiber, polyester, and polyvinylidene difluoride membranes that have been used [31]. Six different papers were selected to optimize the paper substrates. As shown in Figure 3B, the fluorescence intensity of SX18, BT53 and VL78 were stronger without elution, but after elution the fluorescence intensity decreased by 77.37%, 44.57% and 66.73%, respectively, indicating that the three kinds of paper had poor binding ability with CdSe QDs. Moreover, high background fluorescence (λ_ex_ = 370 nm) was observed near 550 nm, which could be attributed to the addition of fluorescent agents in the paper manufacturing process to achieve ideal whiteness [32]. However, SB08, CB06 and RB45 showed low fluorescence value near 550 nm (Figure 3A). Furthermore, CB06 showed relatively reasonable fluorescence intensity before and after elution of CdSe QDs. Consequently, CB06 was selected as the ideal paper substrate for subsequent experiments.

### 3.3. Possible Binding Mechanism between CdSe QDs and Paper Substrate

In order to study the interaction between CB06 and CdSe QDs, urea, SDS and NaCl solution were used to detect the hydrogen bond, hydrophobic interaction and electrostatic interaction, respectively. As shown in Figure 3C, the combination between CdSe QDs and CB06 was mainly dominated by hydrogen bond and hydrophobic interaction, which was different from our previous work. This may because the used CdSe QDs was synthesized in the oil phase, and there may be hydrophobic interactions and hydrogen bond interactions between the oleic acid/oleamine ligands on the surface of CdSe QDs and the paper substrate.

### 3.4. Selection of Functional Monomers

As a simple and practical method to study the interaction between molecules, UV-vis spectroscopy was used to explore the interaction between template molecules and functional monomers [33]. Here, we used dimethoate as template and silanization reagent as functional monomer. According to Figure 3D, after adding MPTMS and APTES, the absorption value of dimethoate decreased by 54.60% and 29.35%, respectively. When interacted with MPTMS and APTES in different molar ratios, it could be seen from the Figure 3E,F that the absorption value of dimethoate decreased, accompanied with a red shift of the absorption peak at the same time, which provided evidence for the interaction between silanization agents and dimethoate. More importantly, the influence of MPTMS on dimethoate was more obvious. Therefore, it seemed like that the MPTMS was an ideal functional monomer for dimethoate.

**Figure 4:**
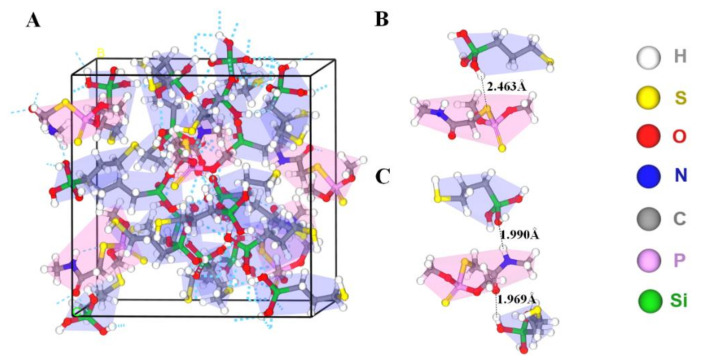
(**A**) Prepolymer system of dimethoate (red background) and MPTMS (blue background). (**B**,**C**) Two different kinds of hydrogen bonding between dimethoate and MPTMS.

**Figure 5:**
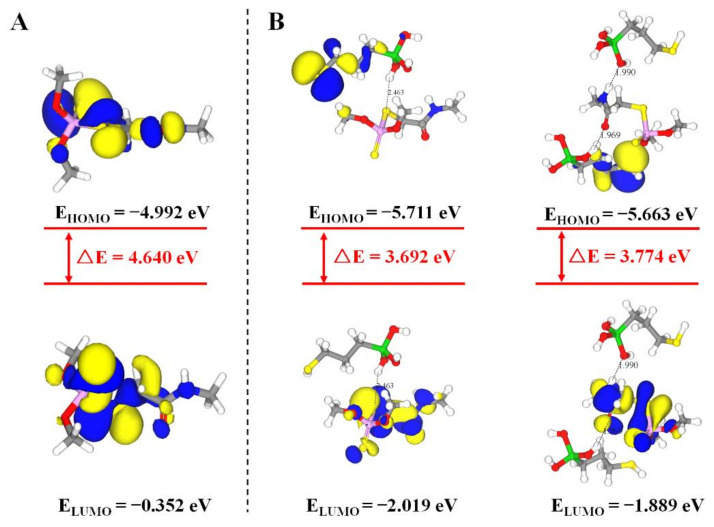
HOMO–LUMO energy gaps and interfacial plots of the orbitals for dimethoate (**A**) and dimethoate–MPTMS (**B**).

In the non-covalent imprinting process, the interaction between the functional monomers and the templates via hydrogen bond is the most common [15]. The Si-OH generated after the hydrolysis of the silanization reagents could bond with the dimethoate molecules through the hydrogen bond. During the elution process, acetic acid was used to break the hydrogen bond between them, so the dimethoate molecules would be eluded from the molecularly imprinted polymers. Consequently, the spatial structures and binding sites matching with the template molecules would be formed on the surface of the paper substrate, which gave the ability of selective recognition of template molecules. In view of that, Materials Studio 2019 was used to simulate the interaction between MPTMS and dimethoate. On the basis of previously published reference [34], molecular simulation was conducted by appropriate modification. The ratio of dimethoate:MPTMS was 5:20 and the PCFF force field was employed. The optimized structure of molecular simulation dynamics is shown in Figure 4. The results show that the prepolymer formed by dimethoate molecules and MPTMS contained a large number of hydrogen bonds. Among them, the H atom on Si-OH generated after the hydrolysis of MPTMS formed C-S⋯H and C=O⋯H hydrogen bonds with C-S and C=O respectively in dimethoate molecules, and the O atom on Si-OH and N-H in dimethoate molecules could form Si-O⋯H-N hydrogen bonds. The molecular simulation was used to verify the electrostatic potential field on the surface of the individual MPTMS and dimethoate (Figure S1). The electron density analysis result showed that electron clouds overlapped between MPTMS and dimethoate, which further confirmed the formation of hydrogen bonds in the system (Figure S2). The positions of HOMO and LOMO orbitals changed when dimethoate molecules combined with MPTMS in two different ways. In addition, compared with dimethoate, the HOMO-LUMO energy gaps of dimethoate–MPTMS complex were smaller (Figure 5). The results indicated that there was electron transfer during the binding process.

### 3.5. Characterization of Imprinted Paper Chip

Firstly, the structure of paper substrates at different periods was observed by SEM. As shown in Figure S3A, the interior of CB06 was crisscrossed, presenting a smooth and clean glass fiber strip structure. After grafting with CdSe QDs, paper@CdSe QDs was further observed (Figure S3B). Since the particle size of CdSe QDs was several nanometers, compared with CB06, the grafting of CdSe QDs did not cause further changes in the microstructure of the paper. For MIPs, SEM image clearly showed that the fiber surface became rough after modification (Figure S3C).

**Figure 6:**
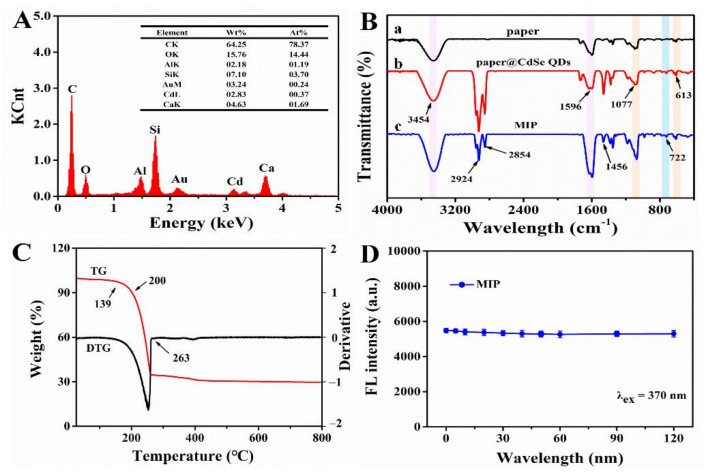
(**A**) EDS image of imprinted paper chip. (**B**) FT-IR spectra of (a) CB06 paper substrate, (b) substrate@ CdSe QDs, (c) imprinted paper chip. (**C**) TG and DTG curves for imprinted paper chip. (**D**) Fluorescence stability of imprinted paper chip.

**Figure 7:**
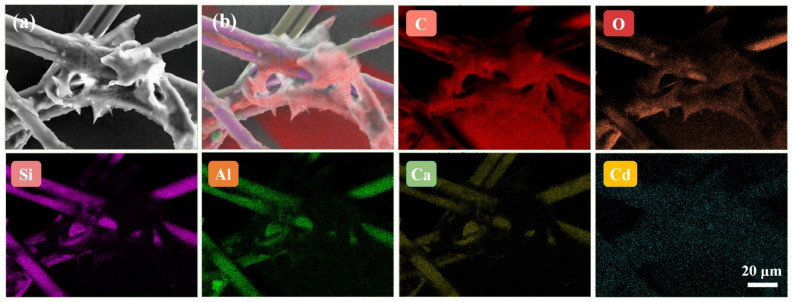
SEM image of the imprinted paper chip (**a**), composite EDS elemental mapping image of six elements (**b**) and corresponding EDS elemental mapping images of C, O, Si, Al, Ca and Cd in imprinted paper chip.

Secondly, EDS elemental mapping was used to analyze the main elements and their distribution of MIPs (Figure 6A and Figure 7). In order to improve the electrical conductivity of the sample, we sprayed gold onto the surface of the paper, which led to the presence of Au element. As for O, Si, Al and Ca elements, they were derived from glass fiber so their distributions were consistent its morphology. In addition, the reason why C element existed was that, on one hand, the conductive adhesive used for sample preparation contained C element, and on the other hand the CdSe QDs used in the experiment were synthesized in the organic phase. As a result, part of the C element was deposited on the glass fiber during the synthesis of MIPs. Compared with pristine paper, MIPs had the characteristic peak of Cd element (0.37%), which only existed in CdSe QDs. Meanwhile, we also observed the Cd element on glass fiber with relatively uniform distribution. These results confirmed that CdSe QDs had been successfully deposited on CB06 paper substrate.

Thirdly, the changes of groups on the paper substrate surface were observed by FT-IR during modification. In Figure 6B, the peaks at 3454 cm^−1^ and 1596 cm^−1^ were caused by N-H bending vibration and gradually increased from curve a to c, indicating that -NH_2_ was successfully connected to the paper substrate, which came from CdSe QDs and NH_3_·H_2_O. A wide and strong absorption peak of Si-O-Si was observed near 1077 cm^−1^, and a symmetric stretching vibration peak of Si-O appeared at 613 cm^−1^. The characteristic peaks of silicon at these two places demonstrated the existence of SiO_2_, and increased gradually from curves a to c, verifying the successful modification of paper by silanized materials. The peak at 722 cm^−1^ in curves b and c was the characteristic peak of Cd-Se [24], which proved the successful grafting of CdSe QDs. The carboxyl group and amino group could form an amide bond and produced a C-N vibration absorption peak at 1456 cm^−1^ [35]. These results provided further evidence for the successful modification and synthesis of MIPs.

Finally, paper chips are required to be stable in practice. Therefore, thermal and fluorescence stability are key indicators. Based on this, the thermal stability of MIPs was studied. Figure 6C shows the TG and DTG curves of MIPs, which had three obvious quality changes. There was no significant change in the range of 0–139 °C. When the temperature reached 139 °C, the weight began to decrease gradually, and the degradation rate was fastest in the range of 200–263 °C, then tended to reach the plateau after 263 °C. This demonstrated that MIPs had better thermal stability under 139 °C. Afterwards, the fluorescence stability of MIPs was investigated. The fluorescence intensity was recorded after multiple excitations within 2 h (λ_ex_ = 370 nm). The result showed that, under multiple excitations, the emission of MIPs was stable with a relative standard deviation (RSD) of 1.4%; that is to say, MIPs had good fluorescence stability (Figure 6D).

**Figure 8:**
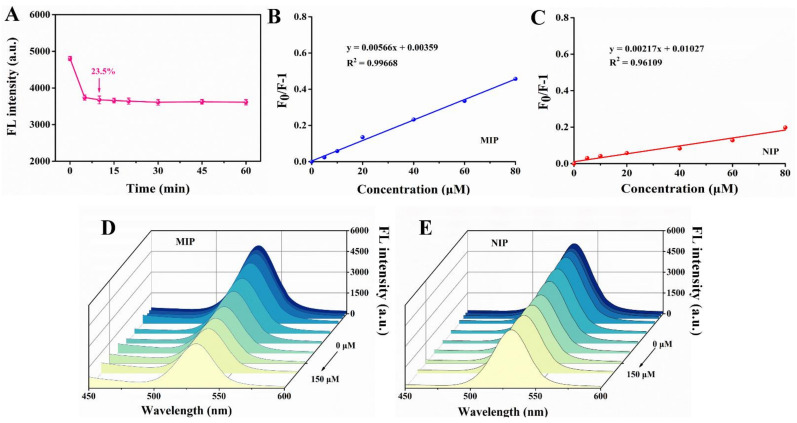
(**A**) Fluorescence response time of imprinted paper chip with 60 μM dimethoate. Linear response of fluorescence change of MIP (**B**) and NIP (**C**). The fluorescence changes of MIP (**D**) and NIP (**E**) at different concentrations of dimethoate (0, 5, 10, 20, 40, 60, 80, 100, 120 and 150 μM).

As we know, one of the characteristics of rapid detection in food safety is the short detection time. In consequence, we studied the response time of MIPs to template molecule. As can be seen from Figure 8A, after adding 60 μM dimethoate solution, the fluorescence of the MIPs decreased by 23.50%, and the fluorescence value tended to be stable after 10 min, so 10 min seemed to be the appropriate reaction time for better accuracy in a short time. Based on this, dimethoate at different concentrations was added to MIPs and NIPs separately every 10 min. The fluorescence intensity of MIPs decreased with the increase of dimethoate content and the linear relationship of MIPs was better than that of NIPs (Figure 8B–E).

**Figure 9:**
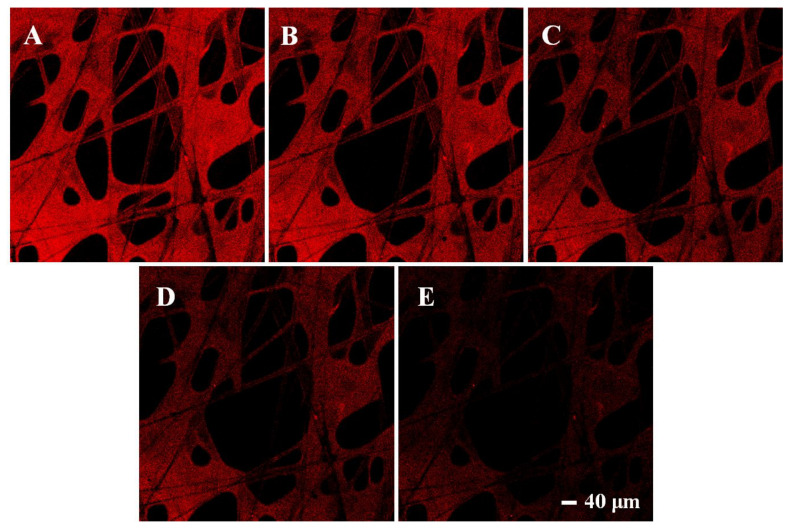
Fluorescence confocal microscope images of CdSe QDs in the imprinted paper chip when dimethoate was added at 0 (**A**), 10 (**B**), 20 (**C**), 40 (**D**), 80 μM (**E**), respectively.

Moreover, fluorescence changes of MIPs with different concentrations were observed under a laser confocal microscope. In order to facilitate observation, the fluorescence color was set as red. As shown in Figure 9, MIPs showed bright fluorescence when no dimethoate was added, which proved the successful graft of CdSe QDs. As the concentration of dimethoate increased gradually (0, 10, 20, 40, 80 μM), the red fluorescence also decreased little by little, which indicated that the presence of dimethoate could indeed suppress the fluorescence of CdSe QDs in MIPs, and as the dimethoate concentration increased, the suppression effect became more obvious. After calculation, the detection range for dimethoate of this method was 0.45–80 μmol/L, and the detection limit was 0.13 μmol/L. These results further confirmed the feasibility of this new rapid detection platform.

### 3.6. Sensitivity and Validation of Method

To explore the selectivity of the sensor, we tested four kinds of OPs with similar structures including trichlorfon, dimethoate, acephate and fenthion. Their chemical structural formulas and the measured responses are shown in Figure S4A. The NIPs displayed no selectivity to all analyzed OPs. As a contrast, MIPs exhibited significant response to dimethoate but similar and weak responses to other OPs, which was attributed to the recognition cavities that were complementary to the dimethoate molecules formed during imprinting process. The result indicated that the selective detection of dimethoate of this sensor could be realized.

In addition, the practical application was studied in soybean samples. The validation of the obtained data was investigated using GC–MS and the results were shown in Table 1. As can be seen in Table 1, no dimethoate at a detectable level was found in soybean samples. According to the report of FAO/WHO, the maximum residue limit for dimethoate in dry beans is 0.7 mg/kg. Thus, more validation experiments were carried out by analyzing the spiked samples. Meanwhile, they were analyzed by the proposed method and the recoveries were calculated. The results showed that all the recoveries were in an acceptable range (97.6–104.1%) and the relative standard deviation (RSD) was 2.6–5.8%. Furthermore, statistical study for the comparison of two methods by ANOVA was performed. As indicated by Table 1, when the dimethoate concentration was low, the difference between these two methods was not significant, but when the dimethoate concentration became larger (20 μmol/L), the difference was significant, indicating that the method we proposed could reduce the error caused by the matrix effect and sample dilution, leading to good applicability for the determination of dimethoate in actual samples.

### 3.7. Possible Quenching Mechanism

The fluorescence quenching mechanism of quantum dots includes static quenching, dynamic quenching, internal filtration effect (IEF), fluorescence resonance energy transfer (FRET), and photoinduced electron transfer (PET) [36].

**Figure 10:**
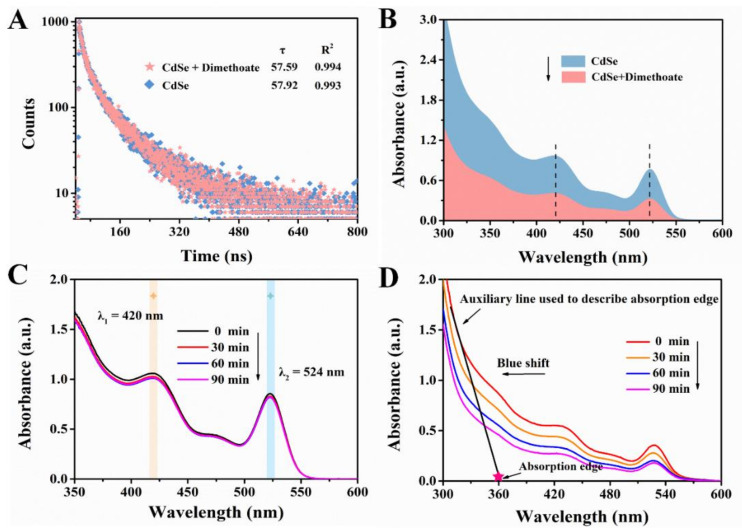
(**A**) Fluorescence decay profile of the CdSe QDs in the absence and presence of dimethoate. (**B**) UV-vis absorption spectra of CdSe QDs in the absence and presence of dimethoate. UV-vis absorption spectra of CdSe QDs without Cu^2+^ (**C**) and with Cu^2+^. (**D**) Treatment with different time of irradiation by ultraviolet lamp.

There are two quenching types in characterizing the mechanism of the interaction between quencher and fluorescence substance: static and dynamic quenching, which can be distinguished by whether the quencher affects the lifetime of the fluorescent substance [37]. Static quenching refers to the formation of non-fluorescent complexes between the quencher and the fluorescence substance. As a result, in the process of static quenching, the existence of the quencher does not affect the fluorescence lifetime of fluorescence substance. On the contrary, dynamic quenching is caused by the collision between fluorescence substance and quencher, which usually tends to change the lifetime of the fluorescence substance [38]. In view of this, we performed the lifetime experiment to investigate the fluorescence attenuation properties of CdSe QDs in the presence and absence of dimethoate. As indicated by Figure 10A, the fluorescence lifetime of CdSe QDs was 57.92 ns, and after treatment with dimethoate the fluorescence lifetime was 57.59 ns. Thus, it could be preliminarily considered that the possible quenching mechanism was static quenching since the lifetime was almost unchanged. Furthermore, we also tested the changes in UV-vis spectra of the system after adding dimethoate. The absorption intensity of CdSe QDs decreased when dimethoate existed (Figure 10B). Accordingly, we speculated that non-fluorescent complexes were formed between dimethoate and CdSe QDs [7].

We could also determine whether the fluorescence quenching was static or dynamic by comparing the value of the quenching constant (Kq) [39]. The Kq can be calculated by the equations as follows:

Kq = K_SV_/τ_0_, (6)

F_0_/F = 1 + K_SV_ [C], (7)

where τ_0_ is fluorescence lifetime without quencher, K_SV_ is the Stern-Volmer quenching constant, which is the slope of linear equation, F_0_ and F are the fluorescence intensities in the absence and presence of quencher, and C is the concentration of the quencher. After calculation, the value of Kq was approximately 9.9 × 10^10^ L·mol^−1^·s^−1^, which was greater than the maximum value of Kq in dynamic quenching. This result further proved the possibility of static quenching [40]. As mentioned above, the fluorescence quenching mechanism between CdSe QDs and dimethoate may belong to the static quenching.

Additionally, quantum dots tend to exchange electrons or energy with complementary partners (acceptor or donor) when in the excited state, thus leading to the quenching of fluorescence [41]. In order to further explore the mechanism of fluorescence quenching of CdSe QDs, the photolysis experiment of CdSe QDs was carried out, and the absorption spectra were tracked. The results were shown in Figure 10C,D, from which it can be seen that CdSe QDs had good stability without Cu^2+^, and its absorption intensity did not change significantly after irradiation by ultraviolet lamp. However, with the existence of Cu^2+^, CdSe QDs gradually degraded, indicated by the extension of irradiation time, the absorption edge of CdSe QDs’ gradual blue shift and the absorption intensity gradually decreasing. The results showed that the fluorescence quenching of CdSe QDs was caused by PET.

## 4. Conclusions

In conclusion, a low-cost, rapid and accurate detection method for dimethoate was established for inhibition effect on the fluorescence signal of oil-phase CdSe QDs. The introduction of microfluidic technology endowed the detection platform with better portability and user-friendly operation. It is worth noting that CB06, a kind of glass fiber paper, was selected as the novel substrate of paper chips owing to its excellent binding ability with CdSe QDs, and the binding mechanism between CdSe QDs and paper chip substrate was discussed in detail. In addition, the surface imprinting technique successfully constructed a specific analytical platform, in which dimethoate molecules can be selectively adsorbed, and then inhibited the fluorescence of CdSe QDs by static quenching and PET. More importantly, in the process of molecular imprinting, the interactions between different functional monomers and dimethoate were studied. The results showed that MPTMS was an ideal functional monomer because of its stronger interaction with dimethoate molecules, which was also theoretically supported by molecular simulation. Finally, the sensor was applied to the analysis of dimethoate in soybean samples to evaluate practicability, and the accuracy of results was verified by GC-MS. On the basis of these results, it can be concluded that the proposed approach was applicable to detect dimethoate in routine food samples in order to guarantee food safety.

## Figures and Tables

**Table 1:** Spiked recoveries and relative standard deviations (RSD, %, *n* = 3) for the determination of dimethoate in soybean with microfluidic paper chips and GC-MS.

Sample	Spiked (μmol/L)	MIPs			GC-MS
		Found (μmol/L)	Recovery	RSD (%)	Determined (μmol/L)
Soybean	0	ND	–	–	–
	5.00	5.08 ± 0.29 ^1^ a	101.7 ± 5.9	5.8	4.47 ± 0.08 a
	10.00	10.41 ± 0.27 a	104.1 ± 2.7	2.6	8.76 ± 0.11 a
	20.00	19.52 ± 1.05 a	97.6 ± 5.3	5.4	17.61 ± 0.13 b

^1^ Data are presented as means ± standard deviation with three replications. Statistical analysis between the two methods is by ANOVA. Different letters (a and b) show a significant difference in the same row (*p* < 0.05).

## Data Availability

Data are contained within the article and Supplementary Material.

## Supplementary Materials

The following are available online at https://www.mdpi.com/article/10.3390/foods10112810/s1, Figure S1. Electrostatic potential field on the surface of dimethoate and MPTMS molecules. Figure S2. Electron density analysis of prepolymer formed by dimethoate and MPTMS. Figure S3. SEM images of (A) untreated CB06 paper substrate, (B) paper@ CdSe QDs, (C) imprinted paper chip. Figure S4. (A) Selectivity of MIPs and NIPs for dimethoate and other analogues (acephate, trichlorfon and fenthion), (B) Chemical formulas of dimethoate, acephate, trichlorfon and fenthion.
