# Supplementary material for: Rapid Detection of Dimethoate in Soybean Samples by Microfluidic Paper Chips Based on Oil-Soluble CdSe Quantum Dots
Source: Foods. 2021 Nov 15;10(11):2810. doi: 10.3390/foods10112810 (PMC8619599; doi:10.3390/foods10112810)
Supplement: Supplementary file 1 [file foods-10-02810-s001.zip › foods-1434083-supplementary.pdf]

Supplementary materials

Rapid Detection of Dimethoate in Soybean Samples by Microfluidic Paper Chips Based on Oil-Soluble CdSe Quantum Dots

Xinpeng Yan [1], Zhong Zhang [1,2], Runguang Zhang [1,2,*], Tian Yang [1], Guoying Hao [1], Li Yuan [1] and Xingbin Yang [1,2]

[1] Shaanxi Engineering Laboratory for Food Green Processing and Safety Control, Engineering Research Center of High Value Utilization of Western Fruit Resources, Ministry of Education, College of Food Engineering and Nutritional Science, Shaanxi Normal University, Xi'an 710062, China; echoyan@snnu.edu.cn (X.Y.); zzhang@snnu.edu.cn (Z.Z.); tianyang@snnu.edu.cn (T.Y.); haogy@snnu.edu.cn (G.H.); Yuanli112086@snnu.edu.cn (L.Y.); xbyang@snnu.edu.cn (X.Y.)

[2] Xi'an Key Laboratory of Characteristic Fruit Storage and Preservation, Shaanxi Key Laboratory for Hazard Factors Assessment in Processing and Storage of Agricultural Products, Xi'an 710119, China

* Correspondence: sunshine@snnu.edu.cn

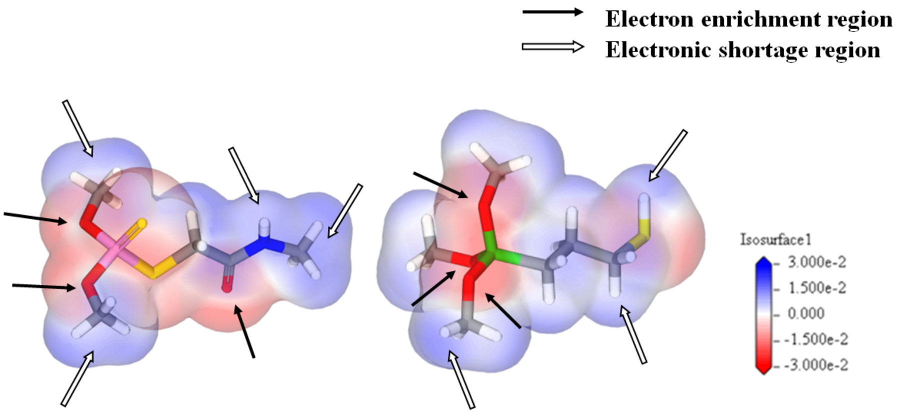

Figure S1. Electrostatic potential field on the surface of dimethoate and MPTMS molecules.

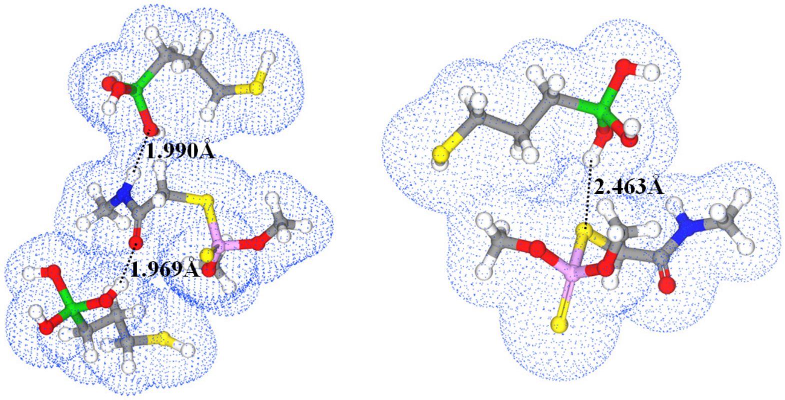

Figure S2. Electron density analysis of prepolymer formed by dimethoate and MPTMS.

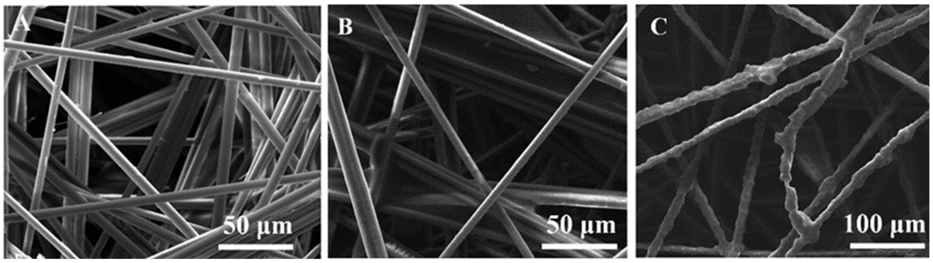

Figure S3. SEM images of (A) untreated CB06 paper substrate, (B) paper@ CdSe QDs, (C) imprinted paper chip.

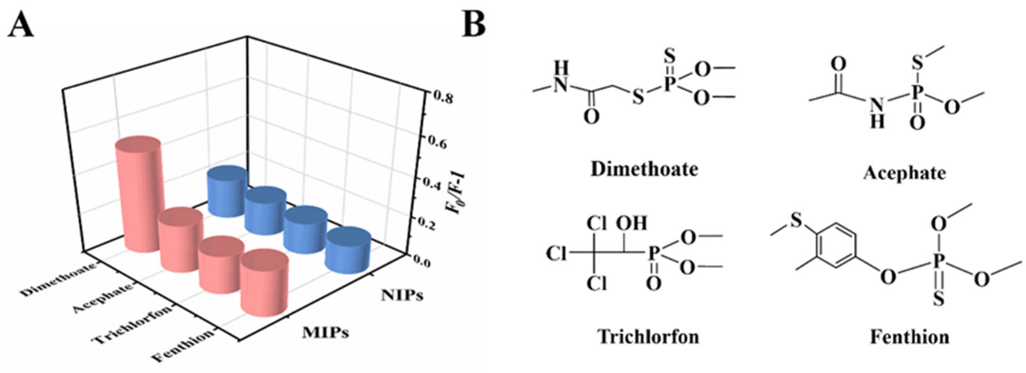

Figure S4. (A) Selectivity of MIPs and NIPs for dimethoate and other analogues (acephate, trichlorfon and fenthion) (B) Chemical formulas of dimethoate, acephate, trichlorfon and fenthion.
